# Cardiac hormones are potent inhibitors of secreted frizzled-related protein-3 in human cancer cells

**DOI:** 10.3892/etm.2012.806

**Published:** 2012-11-12

**Authors:** WILLIAM P. SKELTON, MICHELLE SKELTON, DAVID L. VESELY

**Affiliations:** 1 Departments of Medicine, Molecular Pharmacology and Physiology, James A. Haley Veterans Administration Medical Center and University of South Florida Morsani Health Sciences Center, Tampa, FL 33612, USA

**Keywords:** secreted frizzled-related protein 3, cardiac hormones, colorectal adenocarcinoma, pancreatic carcinoma, renal adenocarcinoma

## Abstract

Secreted frizzled-related proteins (sFRPs) are secreted glycoproteins involved in neoplastic growth. Four hormones synthesized in the heart, namely vessel dilator, atrial natriuretic peptide (ANP), kaliuretic peptide (KP) and long-acting natriuretic peptide (LANP), have anticancer effects both *in vitro* and *in vivo*. These heart hormones were evaluated for their ability to inhibit sFRP-3, which is associated with tumor invasiveness, in human pancreatic cancer, colorectal cancer and renal adenocarcinoma cell lines. Vessel dilator, KP, ANP and LANP maximally reduced the concentration of sFRP-3 by 83%, 83%, 84% and 83%, respectively (each at P<0.0001), in the human colorectal adenocarcinoma cells. In the human pancreatic carcinoma cells, the concentration of sFRP-3 was maximally reduced by 77%, 77%, 77% and 78% (each at P<0.0001) secondary to treatment with vessel dilator, KP, ANP and LANP, respectively. In the human renal adenocarcinoma cells, the sFRP-3 was maximally reduced by vessel dilator, KP, ANP and LANP by 68%, 66%, 68% and 66% (each at P<0.0001), respectively. The results indicate that these four cardiac hormones are significant inhibitors (up to 84%) of sFRP-3 in a variety of human cancer cells. Furthermore, these data suggest that the metabolic targeting of sFRP-3 by the cardiac hormones contributes to their anti-cancer mechanism(s) of action.

## Introduction

Secreted frizzled-related proteins (sFRPs) are palmitoylated secreted glycoproteins that are involved in cell proliferation and neoplastic growth (1,2). sFRPs consist of ∼300 amino acids and are composed of a cysteine-rich domain (CRD) at their amino terminal ends with 30–50% homology to the active site of the Frizzled receptor (3,4). The CRD of Frizzled serves as the active site for Wnt binding and subsequent signal transduction. This class of CRD is conserved in diverse proteins, including the seven-transmembrane class of tyrosine receptor kinases of the receptor tyrosine kinase-like orphan receptor (ROR) family (5). sFRPs act as extracellular signaling ligands and are able to downregulate Wnt signaling by forming an inhibiting complex with the Frizzled receptors (6). Since Wnt causes cancer cells to grow, it was originally hypothesized that sFRPs are inhibitors of cancer cell growth (7) but a subsequent study revealed that sFRP-3 (also known as FrzB) is present at high levels in metastatic renal cancer tissues (8). This study also demonstrated that sFRP-3 promotes invasion by renal cancer cells (8). sFRPs have been linked to tumor-promoting activities in other types of cancer (9). The elevated levels of sFRP-3 in various types of cancer suggest that it may be a valuable therapeutic target (7).

Four endogenous cardiac hormones [vessel dilator, kaliuretic peptide (KP), atrial natriuretic peptide (ANP) and long-acting natriuretic peptide (LANP)] have anticancer effects *in vivo*(10–12), and *in vitro* have been reported to decrease the numbers of human renal carcinoma cells by up to 81% (13), human colorectal cancer cells by 89–97% (14) and pancreatic cancer cells by up to 65% (15). The present investigation was designed to determine whether the four cardiac hormones inhibit sFRP-3 in human renal carcinoma, human pancreatic cancer and human colorectal cancer cells as part of their anti-cancer mechanism(s) of action. The results showed that each of the four cardiac hormones potently inhibited sFRP-3 in the three different types of cancer.

## Materials and methods

### Cardiac hormones

The four cardiac hormones were obtained from Phoenix Pharmaceuticals, Inc. (Belmont, CA, USA).

### Human colorectal, pancreatic and renal cancer cells

Human colorectal cancer (ATCC number CCL-225), pancreatic carcinoma (ATCC number CRL-1469, panc-1) and renal adenocarcinoma (CRL-1611) cells were obtained from American Type Culture Collection (ATCC; Manassas, VA, USA). The ATCC authenticated these cell lines and performed the genotype and phenotype evaluations, including DNA profiles (STR) and cytogenetic analyses.

### Culturing of human colorectal adenocarcinoma cells

The propagation of the human colorectal adenocarcinoma cells was performed in Roswell Park Memorial Institute (RPMI)-1640 medium with 2 mM glutamine adjusted with the addition of 1.5 g/l sodium bicarbonate, 4.5 g/l glucose, 10 mM HEPES, 1 mM 90% sodium pyruvate and 10% fetal bovine serum (FBS; Sigma Chemical Co., St. Louis, MO, USA) at a temperature of 37°C with 5% CO_2_ as recommended by the ATCC. Cells were dispensed into new flasks with sub-culturing every 6–8 days. The growth medium was changed every three days.

### Culturing of human pancreatic carcinoma cells

The propagation of the human pancreatic carcinoma cells was carried out in Dulbecco’s modified Eagle’s plus Ham’s F12A 1:1 mixture containing 1.2 g/l sodium bicarbonate (Sigma Chemical Co.) supplemented with 15 mM HEPES and FBS 10% with 5% CO_2_ at a temperature of 37°C, as recommended by the ATCC. Cells were dispensed into new flasks with subculturing every 6–8 days. The growth medium was changed every 3 days.

### Culturing of human renal adenocarcinoma cells

The propagation of the human renal cell adenocarcinoma cells was carried out in Eagle’s Minimum Essential Medium supplemented with 2 mM glutamine adjusted by the addition of 1.5 g/l sodium bicarbonate, 1 mM 90% sodium pyruvate and 10% FBS (Sigma Chemical Co.) with 5% CO_2_ at a temperature of 37°C, as recommended by the ATCC. Cells were dispensed into new flasks with subculturing every 6–8 days. The growth medium was changed every 3 days.

### sFRP-3 ELISA

Analysis of sFRP-3 was carried out using the DuoSet sFRP-3 immunoassay (R&D Systems, Inc., Minneapolis, MN, USA), a 6-hour solid phase ELISA designed to measure sFRP-3 levels in cell culture. In this assay, an immobilized capture antibody specific for sFRP-3 binds to sFRP-3 using a standard streptavidin conjugated to horseradish peroxidase. This ELISA specifically recognizes sFRP-3 without cross-reactivity or interference with FRP-1, FRP-4 and sFRP-2. The sFRP-3 ELISA was calibrated against a highly purified NSO-expressed recombinant human sFRP-3 (R&D Systems, Inc). The standard curve for this assay was calculated using a four-parameter logistic (4-PL) curve fit.

### sFRP-3 research protocol

The human colorectal cancer, pancreatic carcinoma and renal adenocarcinoma cells were subcultured for 24 h, then ∼5,000 cells of each line in 50 μl of their respective media were seeded in 96-well plates with 50 μl media containing 10 μM, 1 μM, 100 nM, 10 nM, 1 nM and 100 pM concentrations of each of the four cardiac hormones separately (i.e. six concentrations of four cardiac hormones measured six times at each concentration; n=6 for each concentration). Standards from R&D Systems were diluted using Reagent Diluent and added to blank wells to serve as reference points of known sFRP-3 concentrations. In this assay, absorbance was examined at a 540 nm wavelength using a 96-well Gen5, Synergy Mx microplate reader (BioTek, Winooski, VT, USA) set according to the parameters recommended by the manufacturer. There were 32 controls for each cell line (n=32) and six experimental determinations for each of the six concentrations of the four cardiac hormones in the three cancer cell lines (n=6).

### Statistical analysis

Data are expressed as the means ± SEM. The statistical analyses of the data were performed using a Student’s t-test for unpaired values. P<0.05 was considered to indicate a statistically significant difference.

## Results

### Inhibition of sFRP-3 in human colorectal cancer cells

**Figure 1.**
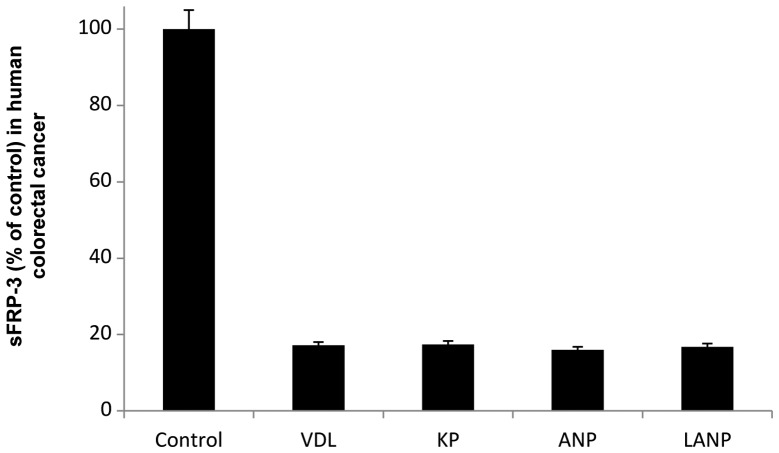
Vessel dilator (VDL), kaliuretic peptide (KP), atrial natriuretic peptide (ANP) and long-acting natriuretic peptide (LANP) maximally reduced the level of secreted frizzled-related protein 3 (sFRP-3) (in pg/ml) in human colorectal cancer cells by 83%, 83%, 84% and 83%, respectively. Each of these reductions was significant at P<0.0001 when evaluated by the Student’s t-test for unpaired values. Each bar represents the mean ± SEM of 32 determinations for the control and 6 determinations for the experimental groups.

An 83% (P<0.0001) reduction of the sFRP-3 level was observed in the human colorectal cancer cells following treatment with 100 nM vessel dilator (Fig. 1). KP and ANP caused maximal reductions of the sFRP-3 level in the human colorectal cancer cells of 83% and 84% respectively, both at a concentration of 100 nM (P<0.0001), while LANP caused a maximal decrease of 83% (P<0.0001) at a concentration of 10 μM (Fig. 1). In the human colorectal cancer cells, each of the cardiac hormones caused a similar significant (P<0.0001) decrease in the sFRP-3 level.

### Inhibition of sFRP-3 in human pancreatic carcinoma cells

**Figure 2.**
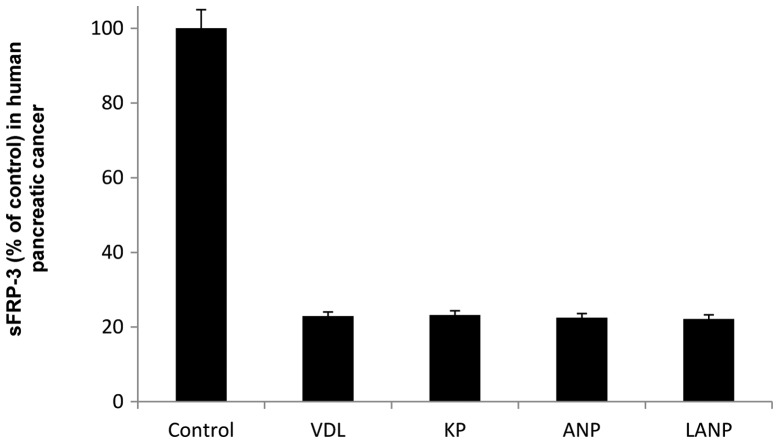
Secreted frizzled-related protein 3 (sFRP-3) in human pancreatic cancer cells was maximally reduced by 77%, 77%, 77% and 78% by vessel dilator (VDL), kaliuretic peptide (KP), atrial natriuretic peptide (ANP) and long-acting natriuretic peptide (LANP), respectively. Each of these reductions was significant at P<0.0001 when evaluated by the Student’s t-test for unpaired values. Each bar represents the mean ± SEM of 32 determinations for the control and 6 determinations for the experimental groups.

Vessel dilator maximally reduced the sFRP-3 level by 77% (P<0.0001) in the human pancreatic cancer cells at its 1 nM concentration (Fig. 2). KP and ANP also maximally reduced the sFRP-3 level in the human pancreatic cancer cells by 77% (P<0.0001), the former at a concentration of 100 pM and the latter at concentrations of 100 nM and 1 nM (Fig. 2). LANP reduced the sFRP-3 level in the human pancreatic cancer cells by 78% at its 1 nM concentration (P<0.0001; Fig. 2). Each of the cardiac hormones had a similar marked ability to reduce the sFRP-3 level in the human pancreatic cancer cells.

### Inhibition of sFRP-3 in human renal adenocarcinoma cells

**Figure 3.**
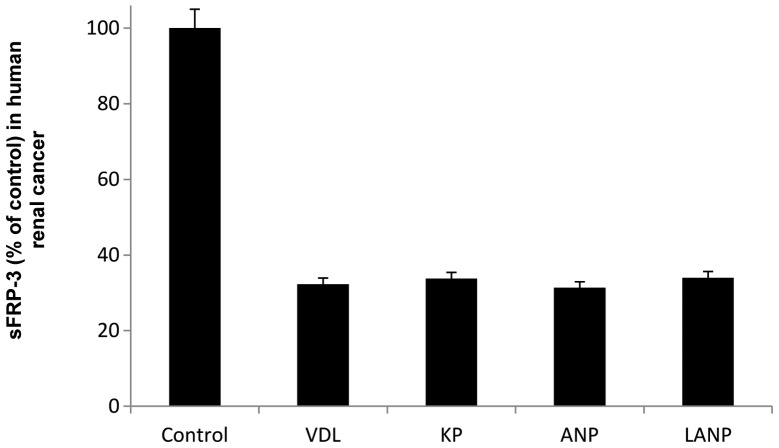
The level of secreted frizzled-related protein 3 (sFRP-3) in human renal adenocarcinoma cells was maximally reduced by 68%, 66%, 68% and 66%, by vessel dilator (VDL), kaliuretic peptide (KP), atrial natriuretic peptide (ANP) and long-acting natriuretic peptide (LANP), respectively. Each of these reductions was significant at P<0.0001 when evaluated by the Student’s t-test for unpaired values. Each bar represents the mean ± SEM of 32 determinations for the control and 6 determinations for the experimental groups.

Vessel dilator decreased the sFRP-3 level in the human renal cancer cells by 68% at a concentration of 1 μM (P<0.0001), and KP maximally reduced the sFRP-3 level by 66% (P<0.0001) at the same concentration. The maximal decrease in the sFRP-3 level of the human renal cancer cells following treatment with ANP was 68% (P<0.0001) at a concentration of 10 nM and following treatment with LANP was 66% (P<0.0001) at a concentration of 100 nM (Fig. 3). In the human renal cancer cells, the abilities of each of the cardiac hormones to inhibit human sFRP were not significantly different as each caused similar significant decreases in the sFRP-3 level. The four cardiac hormones caused similar marked decreases in the sFRP-3 levels of the human colorectal, pancreatic and renal cancer cells (Figs. 1–3).

## Discussion

It has been reported that sFRP-3 promotes renal cancer growth when injected into nude mice (8). sFRPs have also been linked to tumor promotion in other types of cancer (9). It has been suggested (7) that the elevated sFRPs in various types of cancers may be valuable therapeutic targets. The present investigation demonstrates that vessel dilator, KP, ANP and LANP decreased the levels of sFRP-3 by 77–78% in human pancreatic cancer cells, 83–84% in human colorectal cancer cells and 66–68% in human renal cancer cells. These significant reductions of sFRP-3 suggest that it is a target of the four cardiac hormones in a variety of types of cancer. With respect to the mechanism by which the reduction of sFRP-3 levels by the cardiac hormones leads to their anticancer effects, the ability to inhibit sFRP-3, the active CRD of the Frizzled receptor (3), blocks the propagation of the signal responsible for causing cancer cell growth.

It is important to note that the reductions in sFRP-3 levels (up to 84%) are similar in magnitude to the 80% elimination of human pancreatic cancers in mice and 86% elimination of human small-cell lung cancers growing in mice (10,11). The decrease in FRP-3 is also similar in magnitude (% decrease) in cell number of cancer cells *in vitro*(13–15). These observations suggests that sFRP-3 is an important therapeutic target of the cardiac hormones in mediating their anticancer effects (9–11). Furthermore, this target is present in more than one cancer type, and the present study demonstrates that sFRP-3 is a treatment target in human pancreatic, renal and colorectal cancers for each of the four agents evaluated.

## References

[1] Dann CE, Hsieh JC, Rattner A, Sharma D, Nathans J, Leahy DJ (2001). Insights into Wnt binding and signalling from the structures of two Frizzled cysteine-rich domains. Nature.

[2] Malbon CC (2004). Frizzleds: new members of the superfamily of G-protein-coupled receptors. Front Biosci.

[3] Rattner A, Hsieh JC, Smallwood PM, Gilbert DJ, Copeland NG, Jenkins NA, Nathans J (1997). A family of secreted proteins contains homology to the cysteine-rich ligand-binding domain of frizzled receptors. Proc Natl Acad Sci U S A.

[4] Lin K, Wang S, Julius MA, Kitajewski J, Moos M, Luyten FP (1997). The cysteine-rich frizzled domain of Frzb-1 is required and sufficient for modulation of Wnt signaling. Proc Natl Acad Sci U S A.

[5] Xu YK, Nusse R (1998). The Frizzled CRD domain is conserved in diverse proteins including several receptor tyrosine kinases. Curr Biol.

[6] Kawano Y, Kypta R (2003). Secreted antagonists of the Wnt signalling pathway. J Cell Sci.

[7] Bovolenta P, Esteve P, Ruiz JM, Cisneros E, Lopez-Rios J (2008). Beyond Wnt inhibition: new functions of secreted Frizzled-related proteins in development and disease. J Cell Sci.

[8] Hirata H, Hinoda Y, Ueno K, Majid S, Saini S, Dahiya R (2010). Role of secreted Frizzled-related protein 3 in human renal cell carcinoma. Cancer Res.

[9] Rubin JS, Barshishat-Kupper M, Feroze-Merzoug F, Xi ZF (2006). Secreted WNT antagonists as tumor suppressors: pro and con. Front Biosci.

[10] Eichelbaum EJ, Sun Y, Alli AA, Gower WR, Vesely DL (2008). Cardiac and kidney hormones cure up to 86% of human small-cell lung cancers in mice. Eur J Clin Invest.

[11] Vesely DL, Eichelbaum EJ, Sun Y, Alli AA, Vesely BA, Luther SL, Gower WR (2007). Elimination of up to 80% of human pancreatic adenocarcinomas in athymic mice by cardiac hormones. In Vivo.

[12] Vesely DL, Vesely BA, Eichelbaum EJ, Sun Y, Alli AA, Gower WR (2007). Four cardiac hormones eliminate up to two-thirds of human breast cancers in athymic mice. In Vivo.

[13] Vesely BA, Eichelbaum EJ, Alli AA, Sun Y, Gower WR, Vesely DL (2006). Urodilatin and four cardiac hormones decrease human renal carcinoma cell numbers. Eur J Clin Invest.

[14] Gower WR, Vesely BA, Alli AA, Vesely DL (2005). Four peptides decrease human colon adenocarcinoma cell number and DNA synthesis via cyclic GMP. Int J Gastrointest Cancer.

[15] Vesely BA, McAfee Q, Gower WR, Vesely DL (2003). Four peptides decrease the number of human pancreatic adenocarcinoma cells. Eur J Clin Invest.

